# The First Inoculation for Small-Pox

**Published:** 1869-03

**Authors:** 


THE FIRST INOCULATION FOR SMALL-POX.—Cotton Mather, "the distinguished divine," introduced inoculation for small-pox in the 18th century. He accidentally came upon an account of inoculation as practiced in Turkey, and bored physicians with the scheme for a long time unsuccessfully. On the 27th day of June, 1721, Dr. Labdiel Boylton inoculated his only son for small-pox.

